# Esophageal atresia type C with overlapping long upper pouch: A rare variant

**DOI:** 10.1016/j.ijscr.2020.06.007

**Published:** 2020-06-11

**Authors:** Samuel Negash, Hiwote Girma, Hanna Getachew Woldeselassie

**Affiliations:** Division of Pediatric Surgery, Department of Surgery, Addis Ababa University, Ethiopia

**Keywords:** Esophageal atresia, Long upper esophageal pouch, Delayed diagnosis, Survival, Case report

## Abstract

**Background:**

Esophageal atresia is one of the most common pediatric surgical conditions with a high mortality in developing countries. The esophagus and trachea have a complex embryologic development leading to a wide spectrum of esophageal atresia anomalies. Long upper esophageal pouch with distal tracheoesophageal fistula is one of these rare varieties. Only a handful of case reports exist on the subject thus far. Most reports are from low income countries.

**Case presentation:**

A neonate referred to our hospital after 2 weeks of life for excessive secretions, persistent respiratory distress and vomiting. Diagnosis of esophageal atresia was overlooked because nasogastric tube was advanced a considerable length. After repeat x-ray and esophagogram, we confirmed the diagnosis of type C esophageal atresia with a long upper esophageal pouch. Due to the dilemma in diagnosis, surgery was delayed after the 3rd week of life. Luckily the child survived even with extremely delayed intervention.

**Conclusion:**

Esophageal atresia can have an uncommon presentation which causes confusion in the diagnosis. Physicians need to be aware of its existence to avoid unnecessary delays in the management. Fortunately, this variant generally seems to have a good prognosis.

## Highlights

• A long upper esophageal pouch extending below T4 level is a rare finding in Esophageal atresia.
• This type of esophageal atresia creates confusion on the traditional NG tube and x-ray test, leading to diagnostic dilemma.
• The surgery is technically simpler in this variant with good outcome.
• Any baby in which the NG tube does not extend below the diaphragm should be considered to have EA until proven otherwise.

## 1 Background

Esophageal atresia (EA) is the most common anomaly of the esophagus. There are 5 major types as described by Gross [1]. Type C esophageal atresia with distal fistula (TEF) is by far the most common type accounting for 80% [2]. It is easy to diagnose by resistance to passing a 10Fr orogastric tube [3]. Characteristically the upper segment ends at the level above the tracheal bifurcation (T4) [2]. Radiographs showing the tube coiled around this site confirm the diagnosis [4].

There is a very rare subtype of type-C EA first reported in 1960 [45]. In this variant the level of the upper esophageal pouch is lower than usual, sometimes reaching as far down as the diaphragm [4]. This causes a diagnostic confusion as the traditional feeding tube test gives the false impression of lying in the stomach [3]. Esophagogram can also be mistaken for esophageal stenosis [6]. Some mandate esophagoscopy and bronchoscopy in such cases [4,7]. Still others have failed to diagnose with bronchoscopy, which led them to perform a gastrostomy with retrograde esophagography [6].

There are only a handful of case reports entailing this rare phenomenon worldwide. We recently came across a similar case in our institution causing a diagnostic dilemma. The surgery was delayed to the 22nd day of life with a surprisingly good outcome. Herein we describe our encounter and discuss relevant literature on the subject. The work has been reported in line with the SCARE criteria [8].

## 2 Case presentation

A female neonate was referred to our center from the rural parts of Ethiopia. The mother had an uneventful antenatal follow-up and no abnormality was detected on ultrasound. The child was born term by spontaneous vaginal delivery. Subsequently she went home and was being breastfed when the mother noticed excessive salivation and vomiting after feeding.

The mother took her to a local hospital where the child was admitted in the NICU for 2 weeks for the treatment of sepsis. She was kept NPO, put on intranasal oxygen, maintenance fluid and intravenous antibiotics. However, the respiratory distress and excessive salivation persisted. Referring physicians reported that feeding tube was able to pass down and the x-ray of the child was normal. They had attempted trophic feeding through the NG tube but the child persisted to have vomiting. Finally, she was referred to our center with a suspicion of Type E EA (tracheoesophageal fistula without esophageal atresia) on the 16th day of life.

**Fig. 1:**
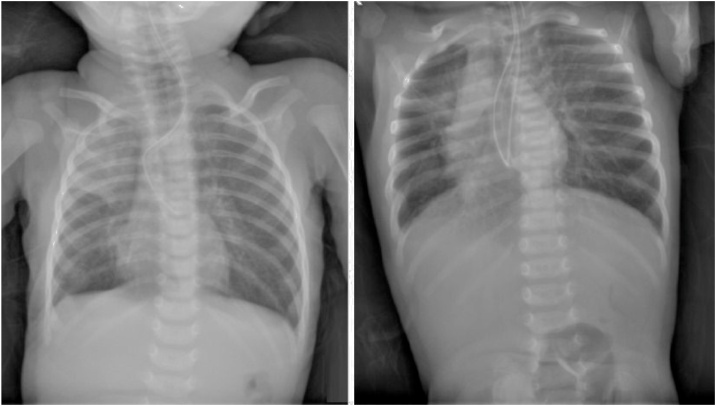
Plain chest x-ray taken on the 16th day of life shows rigid tube with tip at T8 vertebra. Part of the tube is bent with irregular orientation inside the wide esophagus. Repeat x-ray on the same day also shows coiling of the tube around the same level.

On presentation at our center, the child had excessive secretions coming out of the mouth and nose. She had some respiratory distress requiring intranasal oxygen but the other vital signs were stable. Auscultation of the chest revealed crepitations on the right lung, otherwise there was no cardiac murmur. An 8 French NG tube was able to pass more than 20 cm before it met resistance. X-ray taken showed tip of the feeding tube at T8 level. There was also consolidation of the right upper lobe and gas in the stomach (Fig. 1).

**Fig. 2:**
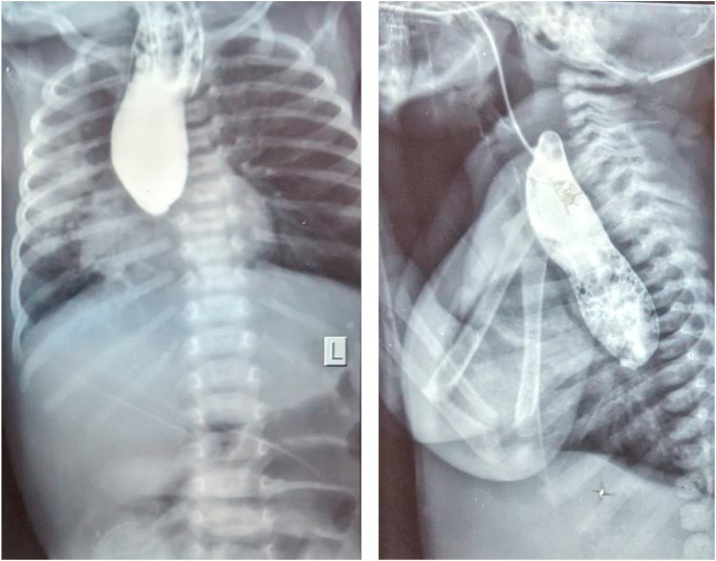
Esophagogram taken on the 20th day of life (AP and lateral views) demonstrate dilated esophagus tapering abruptly at T8 level.

Thereafter a barium swallow study was obtained which demonstrated a long dilated upper esophageal pouch reaching the lower chest (Fig. 2). Surgery was planned with the impression of type C TEF with long upper esophageal pouch. The procedure was delayed by 1 week from the child’s arrival to our center due to resource constraints in obtaining contrast study as well as operating table.

**Fig. 3:**
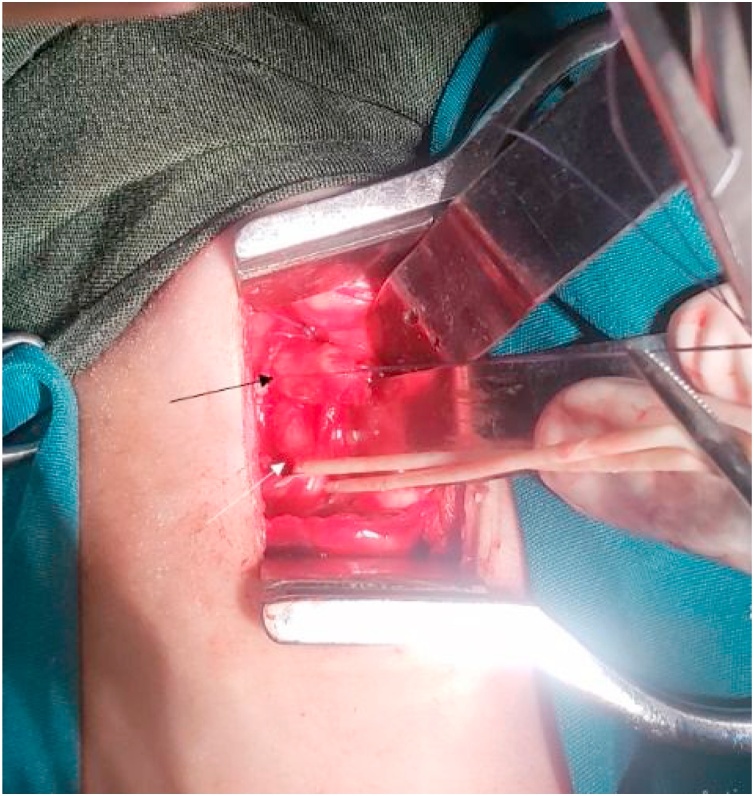
Intraoperative image taken without any mobilization of the esophageal ends. The upper esophageal pouch (black arrow) was found dilated with thick wall and overlapping the lower esophagus (white arrow). The upper esophagus was held with vicryl stay suture to elevate it off the distal esophagus which was identified and held with a loop.

The child was operated onthe 22nd day of life by pediatric surgery fellows supervised by attendings. Bronchoscopy was not performed as it is not routinely practiced during EA surgery in our setting. The child was put in left lateral position after administration of general anesthesia. A right thoracotomy was performed through the 5th intercostal space and thoracic cavity entered through a muscle sparing approach. We found the upper esophageal pouch redundant, overlapping the distal fistula (Fig. 3). After ligating the fistula, end-to end esophageal anastomosis performed without resecting part of the proximal esophagus.

Postoperatively the child didn’t require mechanical ventilation. She had developed postoperative pneumonia requiring intravenous antibiotics and hospital stay of 2 weeks. However, anastomosis healed well and she tolerated full oral feeds. She was finally discharged in excellent condition.

## 3 Discussion and conclusion

Delays diagnosis of tracheoesophageal fistula is a common problem in developing countries, mostly reported from India. It is associated with higher mortality due to malnutrition and pneumonitis [9]. Thus far, there is only 1 report of a survivor operated after the 3rd week (21 days) of life. Gupta reported a surgery delayed to 26th day of life because of delayed presentation to hospital after a home delivery [10]. The case from our report was delayed to the 22nd day of life due to an unusual variant creating a diagnostic dilemma.

Why the upper pouch is markedly elongated in this rare variant is unknown [7]. Older reports have assumed transitory mechanical impingement during aortic development to result in such anomalies [2]. Others suspect it occurs due to the elastic recoil of the dilated upper pouch [4]. We also speculate whether the enlargement was a postnatal occurrence as postulated by John Foker [11]. The late presentation with continued distension of the esophagus could have contributed in our case. However, our review suggests that this is a primary condition as most were diagnosed much earlier (Table 1).

**Table 1:** Case reports of Type C EA with long upper esophageal pouch.

No	Author/Year	Country	Sex/ Age	Level of upper pouch	Associated problems	Surgery	Outcome
1	Dafoe, 1960 [5]	Canada	F, 0 d	Lower 1/3 of thorax	–	Primary anstomosis	Good
2	Roe, 1963 [2]	USA	F, 0d	<1 cm above diaphragm	nil	S-S anastomosis	Good
3	Rathod, 2012 [3]	India	F, 6d	T9	–	Resection, E-E anastomosis	died
4	Rathod, 2012 [12]	India	-, 5d	T8	Nil	E-E anastomosis	Good
5	Kondo, 2015 [6]	Japan	M, 0d	T9	Cardiac, vertebra, ribs	Gastrostomy, E-E anastomosis	Good
6	Yoshu, 2016 [7]	India	M, 0d	T8	Cardiac	Resection, E-E anastomosis	Good
7	Gupta, 2018 [4]	India	M, 1d	T6/T7	Nil	E-E anastomosis	Good
8	Gupta, 2018 [4]	India	M, 9d	T8	Preterm, cardiac, pneumonia	Died before surgery	died
9	Gupta, 2018 [4]	India	M, 3d	T8/T9	Nil	E-E anastomosis	Good
10	our report, 2020	Ethiopia	F, 22d	T8	pneumonia	E-E anastomosis	Good

E-E = end to end, S-S = side to side.

From our literature review we found only 9 previous reports describing this anomaly (Table 1). Half of the reports were from India. Sex distribution is equal. Most diagnostic delays were for a few days, except for this report. Most reported the coiling of feeding tube >18−20 cm from the lower lip. The level of the upper esophageal pouch on x-ray was around T8 in most of the cases.

Intraoperatively, there was no gap between the esophageal ends as long proximal esophagus overlapped with the distal one. As such, the surgery was described as being simple by most. After division of the fistula, different approaches were used to anastomose esophagus. These included side to side anastomosis and end to end anastomosis with or without resection of proximal redundant esophagus. Except for 2 children, all had favorable outcomes.

In conclusion, physicians need to be aware of this rare variant of EA –TEF with an unusual presentation. Having a high index of suspicion will avoid delays in diagnosis and management. Overall, reported cases show a good survival. Our child was extremely fortunate as she survived her condition even with extremely delayed intervention.

## Declaration of Competing Interest

We declare that there is no conflict of interests.

## Funding

This study did not receive funding.

## Ethical approval

Exemption from ethical approval was obtained from the department of surgery ethics and research committee at Addis Ababa University college of health sciences.

## Consent

Written informed consent was obtained from the patient's parent/guardian for publication of this case report and accompanying images. A copy of the written consent is available for review by the Editor-in-Chief of this journal on request.

## Author contribution

SN and HW collected the data and drafted the manuscript. HGW was involved in the conception of the case report and supervised other authors. All authors read and approved the final manuscript.

## Registration of research studies

Registry not required as this is not a first-in-man case report.

## Guarantor

Dr. Samuel Negash.

Division of Pediatric surgery.

Addis Ababa University, Ethiopia.

## Provenance and peer review

Not commissioned, externally peer-reviewed.
